# Effects of Physical Exercise and Motor Activity on Depression and Anxiety in Post-Mastectomy Pain Syndrome

**DOI:** 10.3390/life14010077

**Published:** 2024-01-02

**Authors:** Marco Calapai, Luisa Puzzo, Giuseppe Bova, Daniele Alfio Vecchio, Rosario Blandino, Alessia Barbagallo, Ilaria Ammendolia, Luigi Cardia, Fabrizio Calapai, Mariaconcetta Currò, Giovanni Ficarra, Emanuela Esposito, Fabio Trimarchi, Debora Di Mauro, Gioacchino Calapai, Carmen Mannucci

**Affiliations:** 1 Breast Unit, San Vincenzo Hospital, Azienda Sanitaria Provinciale Messina, 98039 Messina, Italy; marco.calapai@asp.messina.it (M.C.); luisa.puzzo@asp.messina.it (L.P.); daniele.vecchio@asp.messina.it (D.A.V.); rolumic@libero.it (R.B.); 2 Pain Therapy Unit, San Vincenzo Hospital, Azienda Sanitaria Provinciale Messina, 98039 Messina, Italy; bovagiuseppe75@gmail.com (G.B.); barbagalloalessiaanna@gmail.com (A.B.); 3 Department of Clinical and Experimental Medicine, University of Messina, 98125 Messina, Italy; ilaria.ammendolia@unime.it (I.A.); fabrizio.calapai@unime.it (F.C.); mariaconcetta.curro@unime.it (M.C.); giovannificarra96@gmail.com (G.F.); 4 Department of Chemical, Biological, Pharmacological and Environmental Sciences, University of Messina, 98168 Messina, Italy; emanuela.esposito@unime.it; 5 Department of Human Pathology of Adult and Childhood “Gaetano Barresi”, University of Messina, 98124 Messina, Italy; luigi.cardia@unime.it; 6 Genetics and Pharmacogenetics Unit, Policlinico Universitario “G. Martino”, University of Messina, 98125 Messina, Italy; 7 Department of Biomedical and Dental Sciences and Morphological and Functional Imaging, University of Messina, 98100 Messina, Italy; fabio.trimarchi@unime.it (F.T.); debora.dimauro@unime.it (D.D.M.); cmannucci@unime.it (C.M.)

**Keywords:** post mastectomy pain syndrome, pain, physical exercise, depression, anxiety

## Abstract

Background: Chronic post-surgical pain is a condition persisting for not less than 3 months after surgical intervention. It is evaluated that 25–60% of women who underwent breast cancer excision suffer from post-mastectomy pain syndrome, and anxiety, depression, sleep disturbance, and catastrophizing. Physical activity can reduce the risk of chronic diseases and has a good impact on mood and cognitive function. The aim of this study was to estimate the influence of physical activity on the intensity of pain, depression, and anxiety in women who underwent mastectomy for breast cancer removal. Methods: A prospective observational unicentric cohort study was performed. Patients were females who underwent unilateral or bilateral mastectomy. The Numerical Rating Scale (NRS) was used to measure pain intensity, Beck’s Depression Inventory (BDI) for depression, and Generalized Anxiety Disorders-7 (GAD-7) for anxiety evaluation. Physical activity was assessed by the International Physical Activity Questionnaire (IPAQ). Interleukin (IL)-17, IL-1β, cortisol, adrenocorticotropic hormone (ACTH), and brain-derived neurotrophic factor (BDNF) were also evaluated in the blood of patients. All evaluations were assessed 3 and 6 months after the surgery. Results: Adequate physical activity reduced the intensity of pain, depression, and anxiety symptoms in women affected by post-mastectomy pain syndrome. Moreover, adequately active women showed a reduction in biomarkers of inflammation, cortisol, ACTH, and an increase of BDNF. Conclusions: Our results suggest that physical activity can improve the quality of life, reduce the intensity of pain and inflammatory markers, and be useful in the reduction of associated anxiety and depression.

## 1. Introduction

Chronicization of pain frequently occurs after surgery, often leading to functional limitations and psychological disorders with a negative impact on quality of life [1].

Chronic post-surgical pain (CPSP) was first defined as “pain that develops after surgical intervention and lasts at least 2 months; other causes of pain have to be excluded, in particular, pain from a condition existing before the surgery” [2]. An updated definition of CPSP was later proposed as “pain persisting at least 3 months after surgery, that was not present before surgery, or that had different characteristics or increased intensity from preoperative pain, localized to the surgical site or a referred area, and other possible causes of the pain were excluded (e.g., cancer recurrence, infection)” [3]. 

Chronic pain persisting after mastectomy is a major individual and public health problem. The etiology of persistent pain after mastectomy is still unclear because of its possible multifactorial nature [4,5,6], with a partial neuropathic origin [7]. While surgical factors, including axillary lymph node dissection and reconstruction, have been postulated as important risk factors for chronic pain, many studies do not fully support this association. Adjuvant treatment such as radiation, chemotherapy, and hormone therapy have also been occasionally associated with persistent pain consequent to mastectomy [8,9,10].

Psychosocial factors, such as anxiety and catastrophizing, are being revealed as crucial contributors to individual differences in pain processing and outcomes. Some researchers have reported associations between the development of persistent pain, catastrophizing, depression, psychological distress, and reduced physical activity [11,12,13,14,15]. This condition may lead to disability, worsening individual quality of life [16]. Due to the potential benefits induced by physical activity, the American Cancer Society recommends beginning it as soon as possible for people diagnosed with cancer [17].

A recent meta-analysis indicates anxiety as the main psychological risk factor for raising CPSP (and to a lesser degree, depression, catastrophizing, kinesiophobia, and impaired self-efficacy) [18]. Furthermore, many other studies have proven that neuroinflammation is involved in multiple steps of chronic pain, promoting central [19] and peripheral sensitization [20]. Moreover, the relationship between depression, anxiety, and inflammation has been proposed by several works, indicating that levels of inflammatory markers such as C-reactive protein, Interleukin-(IL)6, IL-1β, IL-17, and brain-derived neurotrophic factor (BDNF) are modified in people with depression and anxiety [21,22,23].

It is well-documented that exercise and physical activity undertaken before or after a cancer diagnosis reduce the risk of tumor recurrence and improve overall health, thus increasing survival [24,25,26]. In oncologic patients, exercise and physical activity accelerate the recovery of functional capacities, reduce lymphedema, and increase overall energy levels, thus increasing strength and flexibility, reduce fatigue, improve pain symptoms, and enhance the physical capability to carry out daily activities and reduce the risk of chronic diseases [27,28].

It has been shown that physical activity could inhibit the release of inflammatory cytokines and has beneficial effects on the immune system, influencing the intensity of pain as perceived by patients [29,30,31].

Moreover, several studies suggest that physical activity improves anxiety, sleep, and mood disorders such as depression by modulation of cortisol levels and increases in Brain-Derived Neutrophic Factor (BDNF) levels [32,33]. 

In light of the above-exposed concepts, the objective of this study was to assess the effects of physical activity on the intensity and interference of chronic pain in daily activities and its effect on depression and anxiety in patients who underwent mastectomy.

## 2. Materials and Methods

### 2.1. Study Design

A prospective observational unicentric cohort study was conducted. The population of the study was represented by female patients who underwent unilateral or bilateral mastectomy due to the removal of stage II and III breast cancer, and who had not yet been subjected to breast reconstruction, chemotherapy, or radiation, aged 18 years or over. Pain evaluation for each participant in the study was assessed at 3 and 6 months after surgery through the verbal administration of the Numerical Rating Scale (NRS). Beck’s Depression Inventory (BDI) and General Anxiety Disorders-7 (GAD-7) were used for depression and anxiety assessment, respectively. Physical activity was measured with the International Physical Activity Questionnaire (IPAQ). At the same timepoints, the following blood biomarkers associated with inflammation, depression, and anxiety were evaluated: Interleukin (IL)-17, IL-1β, cortisol, adrenocorticotropic hormone (ACTH), and brain-derived neurotrophic factor (BDNF).

### 2.2. Participants

Enrollment was performed between April and October 2023 at the Breast Unit of San Vincenzo Hospital of Taormina in collaboration with the Azienda Ospedaliera Universitaria (AOU) Policlinico “G. Martino” of Messina, Italy. Inclusion criteria were women aging over 18 years with a prior diagnosis of Phase II or III breast cancer who had undergone mastectomy for cancer removal 3 months earlier.

Exclusion criteria considered were: chemotherapy and radiation throughout the 6 months after surgery, anamnesis including other cancer types, immune system disorders (multiple sclerosis, HIV, lupus), and fresh flu symptoms (cough, fever). Women taking anxiolytic and/or antidepressant and anti-inflammatory drugs in the 15 days before recruitment were also excluded. Patients with breast cancer at Stages 0 and I were not included because of the possible and frequent lack of pain. Women with cancer at Stage IV were not included, as pain can derive from metastases. Women reporting pain prior to surgery and those affected by other types of tumors or other diseases characterized by chronic pain were also excluded. All patients were asked to sign the informed consent form to be included in the study. The study was approved by the Ethics Committee of AOU Policlinico “G. Martino”: Approval Number: Prot. 70-23, 18 April 2023, Board Name: Comitato Etico Interaziendale Messina. The trial was conducted according to the ethical principles of the Declaration of Helsinki, and Good Clinical Practice principles were adopted. To enroll subjects in the study, sample size was calculated using Clinicalc.com (https://clincalc.com/, accessed on 18 April 2023), (ClinicalTrials.gov Identifier: NCT06123559).

### 2.3. Methodology

#### 2.3.1. Demographic and Surgical Variables

Demographic variables influencing pain-related conditions, including age, marriage, and school level, were considered. The Italian education system comprises primary (five years), secondary (three years), post-secondary (five years), and graduation phases (three-six years). Data about lymph nodes dissection were collected for each participant.

#### 2.3.2. Numerical Rating Scale (NRS)

The Numeric Rating Scale (NRS), commonly used to assess pain severity [34], was administered 3 and 6 months after surgery, as previously described [29].

It is important to establish the degree of change representing clinical improvement. The NRS is a 0–11 points scale with endpoints representing the extremes, meaning no pain (point 0) and the worst possible pain (point 10) [34,35]. The score allows the identification of three cutpoints for three levels of pain severity as follows: ratings of 1–4 correspond to mild pain, 5–6 to moderate pain, and 7–10 to severe pain [36]. Based on the collection of medical history and the evaluation of NRS scores 3 months after surgery, women participating in the study were divided into two groups: the “PMP group” with women with pain totaling ≥5 on the NRS, and the “Non-PMP group” composed of women totaling an NRS score < 5. The NRS was newly self-administered 6 months after surgery.

#### 2.3.3. Beck’s Depression Inventory (BDI)

The BDI questionnaire is one of the most widely used psychometric tests for the evaluation of depression. BDI consists of twenty-one questions about how the subject has been feeling in the last week. Each question has at least four possible answer choices; a score of 0 to 3 is assigned for every answer, and then the total score establishes the severity of depression as follows: 0–9 for normal or minimal depression; 10–18 for mild depression; 19–29 for moderate depression; 30–63 for severe depression [37]. BDI was administered 3 and 6 months after surgery.

#### 2.3.4. Generalized Anxiety Disorders-7

The Generalized Anxiety Disorder Assessment (GAD-7) is a seven-item instrument that is used to measure or assess the severity of generalized anxiety disorder (GAD). Each item asks the individual to rate the severity of his or her symptoms over the past two weeks. 

A score of 0, 1, 2, and 3 is assigned to the response categories, respectively, of “not at all,” “several days,” “more than half the days,” and “nearly every day.” The GAD-7 total score for the seven items ranges from 0 to 21, described as follows: 0–4: minimal anxiety; 5–9: mild anxiety; 10–14: moderate anxiety; 15–21: severe anxiety [38]. GAD-7 was administered 3 and 6 months after surgery.

#### 2.3.5. International Physical Activity Questionnaire (IPAQ)

The International Physical Activity Questionnaire (IPAQ) was used to collect information about self-reported physical activity. It was administered 3 and 6 months after surgery, as previously described [29]. This questionnaire measures the type and amount of physical activity. It assesses the number of days and quantity of time spent on physical activity (PA) of moderate or vigorous intensity, walking for at least 10 min during the last 7 days, and also comprises the time spent sitting during the last week. The IPAQ includes four PA levels (work-related activity, leisure-time activity, transport-related activity, and domestic activities), each with 3 degrees of intensities: walking, moderate, and vigorous. Whole weekly physical activity was evaluated by weighing time consumed in each activity intensity together with its calculated metabolic equivalent energy expenditure (Metabolic Equivalent of Task; MET). According to the answers, patients were classified into three categories: inactive, if presenting a METs less than 700, adequate active women presenting a METs value raging between 700 and 2519, and highly active if presenting METs > 3000 [39].

#### 2.3.6. Haematological Biomarkers Associated with Depressive Disorders, Anxiety, and Inflammation

Serum levels of biomarkers were measured 3 and 6 months after surgical intervention, according to the protocol of ELISA kits. We evaluated the following biomarkers: IL-17, IL-1β, cortisol, ACTH, and BDNF. Blood samples were collected between 8–9 a.m. under fasting conditions. The following kits were used: IL-17 (R&D System; Manufactured and Distributed by: USA R&D Systems, Inc. 614 McKinley Place NE, Minneapolis, MN 55413, USA, Catalog #: D1700); IL1-β (R&D System Catalog #: DLB50); BDNF (R&D System Catalog #: DBD00); ACTH (Novus biologicals; Novus Biologicals USA 10730 E. Briarwood Avenue Centennial, CO 80112, USA; NBP2-66401); and cortisol (Novus biologicals NBP3-18003). 

#### 2.3.7. Statistics

The Mann–Whitney U test or Wilcoxon test was used to evaluate the difference among the groups. Data are presented as mean ± standard deviation, and significance was set with a *p*-value < 0.05. Spearman’s rank correlation was used to correlate biomarkers and NRS or BDI or GAD-7 or IPAQ, and to correlate the scores obtained from the questionnaire with each other. According to the test, the correlation is considered “very weak” for values between 0.00–0.19; “weak” for values between 0.20–039; “moderate” for values between 0.40–0.59; “strong” for values between 0.60–079; and “very strong” for values between 0.80–1.0.

## 3. Results

### 3.1. Participants

One hundred and eighty (180) female patients who underwent mastectomy were selected for the study, and one hundred and sixty (160) women were enrolled in the study. The mean age was 50.34 ± 11.9 years (range 28–72 years; median age 53.5 years). The mean BMI was 21.59 ± 1.49. All the participants were Caucasian.

### 3.2. Numerical Rating Scale Score (NRSs)

Clinical examination and estimation of NRS results collected 3 months after surgery showed that 54.4% (*n* = 87) did not report any significant pain (non-PMP group), while 45.6% (*n* =73) of women recruited for the study manifested PMP Syndrome (PMP group). Participants were divided into two groups, the PMP and non-PMP group. 

In the group of PMP patients, the assessment of NRS performed 3 and 6 months after surgery revealed a statistically significant increase in pain intensity in PMP patients compared with those of the non-PMP group (Table 1).

### 3.3. Demographic and Surgical Variables

Results did not show any statistically significant differences between the PMP and non-PMP group for age, education level, lymph nodes dissection, and marital status (Table 1).

### 3.4. Haematological Biomarkers Associated with Depressive Disorders, Anxiety, and Inflammation

Serum levels of IL-17, IL-1β, cortisol, ACTH, were statistically significantly increased in the PMP group compared to the non-PMP group, while BDNF levels were statistically significantly decreased in the PMP group compared to the non-PMP group (Table 2).

### 3.5. BDI, Anxiety, and Physical Activity

BDI and GAD-7 results showed that 60.2% (*n* = 44) of the PMP group totaled scores associated with depression and anxiety. Specifically, 45.45% showed scores linked to severe depression and anxiety, and 34% showed scores associated with moderate depression and anxiety, while 20.55% showed scores associated with scores associated with mild depression and anxiety. According to the BDI and GAD-7 scores, the PMP group was divided into two subgroups: depression anxiety (DA)-PMP subgroup (women reporting a BDI score ≥ 10 and GAD-7 score ≥ 5); and non DA-PMP subgroup (women reporting a BDI ≤ 9 and GAD-7 ≤ 4) (Table 3).

### 3.6. Biomarkers Related to Depression and Anxiety

IL-17, IL1-β, cortisol, and ACTH levels were significantly increased in the PMP group compared to the non-PMP group. These biomarkers were significantly more elevated in the DA-PMP subgroup in comparison with the non-DA-PMP subgroup, either 3 or 6 months after surgery (Table 2 and Table 3). BDNF levels were statistically significantly reduced in the DA-PMP subgroup compared to the non DA-PMP subgroup (Table 3).

### 3.7. IPAQ Score

Physical activity at IPAQ was evaluated among the 44 PMP women showing anxiety and depression (DA-PMP). According to the IPAQ questionnaire, 20 patients have been categorized as inactive (<700 METs), and 24 as adequately active (>700 METs). 

No active women (>2510 METs) were present in the DA-PMP group (Table 4).

DA-PMP inactive women showed a statistically significant increase in the intensity of pain (*p* < 0.01) and an increase in anxiety and depression scores (*p* < 0.01) compared to adequate active DA-PMP women, either 3 or 6 months after surgery (Table 5). 

IL-17, IL-1-β, cortisol, and ACTH levels were statistically significantly increased in inactive DA-PMP women compared to active DA-PMP women (Table 5), while BDNF was statistically significantly reduced in inactive DA-PMP women compared to active women of the same group (Table 5). 

### 3.8. Spearman’s Correlation

Spearman’s correlation was performed to analyze the relationship between scores obtained with questionnaires NRS, BDI, GAD-7, and biomarkers investigated (IL17, IL1-β, cortisol, and ACTH). 

Results showed at 3 and 6 months from surgery a statistically significant positive correlation (*p* < 0.001), between: NRS and IL-17, IL-1β, ACTH, cortisol, BDI and GAD-7 (3 months: rs = 0.91014; rs = 0.91732; rs = 0.93056, rs = 0.932, rs = 0.93071, rs = 0.90087, respectively; 6 months: rs = 0.89196, rs = 0.89816, rs = 0.88876, rs = 0.88764, rs = 0.90454, rs = 0.89787, respectively); between BDI and IL-17, IL-1-β, cortisol, ACTH and GAD-7 (3 months rs = 0.98157, rs = 0.98892, rs = 0.99802, rs = 0.99799, rs = 0.97374; 6 months: rs = 0.97357, rs = 0.91386, rs = 0.99058, rs = 0.99002, rs = 0.97712, respectively), and between GAD-7 and IL-17, IL-1-β, cortisol, ACTH (3 months rs = 0.96508, rs = 0.97011, rs = 0.97574; 6 months: rs = 0.97357, rs = 0.92559, rs = 0.97136, rs = 0.97136, respectively) (Figures S1–S6).

A statistically significantly negative correlation (*p* < 0.001) has been observed between BDNF and NRS, BDI and GAD-7, respectively, both 3 (rs = −0.88222, rs = −0.93432, rs = −0.92469, respectively) and 6 months (rs = −0.86477, rs = −0.91489, rs = −0.923, respectively) after surgery (Figures S1–S6). 

The IPAQ score showed a statistically significant negative correlation (*p* < 0.001) with NRS, BDI, GAD-7, IL-17, IL-1-β, cortisol, and ACTH 3 months after surgery (rs = −0.92328, rs = −0.98736, rs = −0.97649, rs = −0.9753, rs = −0.98407, rs = −0.99091, rs = −0.99158, respectively) and 6 months after surgery (rs = −0.88621, rs = −0.97879, rs = −0.96285, rs = −0.97583, rs = −0.88928, rs = −0.99309, rs = −0.99281, respectively). On the contrary, the IPAQ score showed a statistically significant positive correlation (*p* < 0.001) with BDNF both at 3 (rs = 0.92741) and 6 months (rs = 0.90672) after surgery (Figures S7 and S8).

## 4. Discussion

As defined by the World Health Organization (WHO), physical activity is considered any bodily movement produced by skeletal muscles that requires energy expenditure (WHO, 2019). Physical activity is referred to all movements, including during leisure time, for transport to get to and from places, or daily activities. Both moderate- and vigorous-intensity physical activity improves mental health and reduces the risk of developing noncommunicable diseases such as heart disease, stroke, diabetes, and cancers [27,28]. Physical activity has also been found to improve memory and concentration, thus favoring the protection of cognitive function in the elderly [40], through the stimulation of blood-transported neurotrophic factors secretion, including BDNF, which is also produced in working skeletal muscles [41].

As recommended, adults (18–64 years) should perform weekly at least 150–300 min of moderate-intensity aerobic physical activity or at least 75–150 min of vigorous-intensity aerobic physical activity; alternatively, an equivalent combination of moderate- and vigorous-intensity activity throughout the week. This recommendation is particularly suggested for people affected by chronic diseases such as hypertension, type 2 diabetes, and HIV [42].

It is evaluated that 25–60% of patients who undergo breast cancer surgical resection suffer from PMP syndrome [8]. Psychosocial factors such as anxiety, depression, sleep disturbance, and catastrophizing have proven to be important contributors to the development of persistent pain. A well-known link between pain and depression suggests that 30–45% of patients affected by chronic pain experience depression [43,44,45,46]. Several studies have suggested a bidirectional relationship between depression and pain, suggesting that depression is a positive predictor of the development of chronic pain, and chronic pain can increase the risk of developing depression. Moreover, depression is considered a moderator of the relationship between pain severity and physical functioning. In this view, pain and depression create a vicious cycle where pain worsens symptoms of depression, and the resulting depression negatively influences feelings of pain [47]. It is known that depressive disorders can occur together with anxiety disorders [48,49], and anxiety symptoms are among the diagnostic criteria for major depressive disorder included in the Diagnostic and Statistical Manual of Mental Disorders 5th edition (DSM-5) [50]. Results obtained from case-control studies suggest that inflammation could be involved in generalized anxiety disorder, indicating that inflammation could increase, subsequent to the development of anxiety disorders [51,52]. 

In this study, 45.6% of a sample of women who underwent surgery for breast cancer manifested PMP after the surgical intervention. A similar proportion was found by other authors, reporting a percentage of 43% of women with PMP still three years after breast tumor resection [52]. In this group, IL-17, IL-1-β, cortisol, and ACTH levels were statistically significantly increased compared to the non-PMP group. These results agree with several studies reporting an increase in inflammatory biomarkers in chronic pain and an inbalance of the hypothalamic–pituitary–adrenal axis [53]. It is also suggested that cortisol release during a stress response can inhibit pain; however, when pain is the stressor, cortisol secretion can intensify pain sensitivity. Moreover, other studies suggest that low cortisol levels can increase the perception of pain in the traumatically injured population [54].

60.2% of PMP woman reported symptoms of anxiety and depression (Table 3). In this subgroup, IL-17, IL-1-β, cortisol, and ACTH levels, evaluated either 3 or 6 months after surgery, were significantly enhanced in DA-PMP women in comparison to non-DA-PMP women (Table 3). BDNF levels were significantly reduced in DA-PMP women vs. to non-DA-PMP women (Table 3) both at 3 and 6 months after surgery.

These results are in accordance with other studies indicating that people with major depressive disorders have lower peripheral and central BDNF levels than non-depressed individuals. Moreover, a negative correlation between blood BDNF and symptom severity has been proposed [55]. 

At the same time points, DA-PMP inactive women showed a statistically significant increase in pain intensity (*p* < 0.01) and an increase of anxiety and depression signs compared to DA-PMP adequately active women (Table 3). 

Physical activity was evaluated among DA-PMP patients by the IPAQ, and the results showed that 55% of this group was classified as physically inactive, while 65.5% of this group was adequately active. As stated by the analysis of the answers obtained from the IPAQ, no fully active woman was detected in this group. Six months after surgery, these results overlapped with those obtained 3 months after surgery. 

Our results showed a decrease in IL-17, IL-1β, cortisol, and ACTH in the group of DA-PMP adequately active women compared with DA-PMP inactive women, and an increase in BDNF levels in the group of PMP-AD adequately active women compared with DA-PMP inactive women. 

There is a great deal of correlational evidence that patients who suffer from anxiety and depression show elevations in circulating levels of cytokines that are pro-inflammatory in nature, such as Tumor necrosis factor alpha, IL-1β, IL-6, and IL-17. Furthermore, these patients have elevated levels of leukocytes, which may be the source of these increased inflammatory cytokines [56,57,58,59]. The study hypothesizes a neuroimmune inflammatory role in the pathogenetic mechanism for psychiatric disorders such as depression and bipolar disorders. This hypothesis correlates psychiatric disorders with the activation of the immune–inflammatory response system, resulting in an increase in pro-inflammatory factors and the activation of the compensatory immunoregulatory response system, playing a negative immunoregulatory effect through T helper activation and T regulatory mechanism and suppression of the immune–inflammatory response system hyperreaction [21].

Moreover, several studies have agreed that one of the most important findings in biological psychiatry is the hyperactivity of the hypothalamic–pituitary–Adrenocortical axis observed in patients with major depression [60]. In fact, it has been suggested that prolonged exposure to stress causes metabolic changes such as hypothalamic–pituitary–adrenal axis activation, with a consequent increase in cortisol release [60].

It is known that regular physical activity improves bodily functions, reducing the risk of developing chronic diseases [27,28]. It has also been suggested that even limited amounts of routine daily activities can reduce the risk of falling [61], developing neurologic diseases, and have positive effects on brain health and cognitive function [62,63].

The beneficial effects of physical activity on brain disorders have been widely studied [64,65,66,67]. It has been hypothesized that physical activity could reduce the risk of neurodegenerative diseases, inhibit cognitive decline, and produce a positive effect on stress, anxiety, and depression [67,68], with consequent amelioration of several biomarkers associated with depressive symptoms by the modulation of hypothalamic–pituitary–adrenal (HPA) axis homeostasis (cortisol, testosterone, and dehydroepiandrosterone), anti-neurodegenerative effects (increase of BDNF and VEGF), monoamine metabolism regulation (increase of serotonin norepinephrine, and dopamine), and neuroimmune system functioning (increase of IL-10, decrease of TNF-α, IL-6, IL-1β, cyclooxygenase-2 [33]). The role of physical exercise in increasing BDNF levels is controversial, with some evidence suggesting that acute or chronic exercise programs are able to increase BDNF levels [68], thus indicating that physical activity can be helpful in treating depression and anxiety, increasing neurogenesis. On the contrary, other recent studies suggest that physical exercise did not increase BDNF levels in patients suffering from major depressive disorders [55]. Our results showed an increase in BDNF levels in the DA-PMP group practicing physical activity. The different result could be due to the nature of depressive or anxiety symptoms, correlated in our study with pain and inflammation. 

In conclusion, our results agree with other studies suggesting positive effects of physical activity on anxiety and depression. In our sample, in women affected by post-mastectomy pain syndrome, physical activity was found to reduce signs of anxiety and depression, along with a reduction in pain and levels of inflammatory cytokines, corstisol, ACTH, and an increase in BDNF. These results suggest that exercise could be effective in reducing inflammatory markers and, consequently, prevent the development of chronic post-mastectomy pain syndrome and associated psychiatric disorders such as anxiety and depression.

## Figures and Tables

**Table 1:** Age, education level, lymph nodes dissection, and marital status in PMP and non-PMP groups.

Group	Age (Years)	Education Level	Lymph Nodes Dissection	Marital Status	NRS
Non-PMP (*n* = 87)	49.83 ± 10.94	Secondary = 10.5% Post-secondary = 30.33% Graduation = 12.22%	31.42%	41.72%	1.25 ± 1.67
PMP (*n* = 73)	50.98 ± 12.23	Secondary = 9.8% Post-secondary = 25.10% Graduation = 13.82%	35.15%	42.15%	5.93 ± 1.25 *

PMP = post mastectomy pain group totalizing an NRS score ≥ 5; non-PMP group post-mastectomy pain group totalizing an NRS score < 5; NRS = Numerical Rating Scale * = *p* < 0.01 vs. non-PMP group.

**Table 2:** Evaluation of the intensity of pain and BDNF, IL-17, IL1-β, ACTH, and cortisol in post-mastectomy pain (PMP) and non-post-mastectomy pain (non-PMP) groups at 3 and 6 months after surgery.

	Non-PMP *n* = 87		PMP *n* = 73	
	3 Months	6 Months	3 Months	6 Months
NRS score (intensity of pain)	1.25 ± 1.67	1.43 ± 1.71	5.93 ± 1.25 *	5.81 ± 1.11 *
BDNF (pg/mL)	6112.14 ± 30.18	6114.36 ± 30.12	4607.46 ± 1014.91 *	4552.85 ± 1027.35 *
IL-17 (pg/mL)	13.26 ± 2.11	12.32 ± 2.06	82.92 ± 29.14 *	83.074 ± 27.17 *
IL1-β (pg/mL)	11.26 ± 6.11	11.81± 5.98	76.54± 14.79 *	75.24± 13.84 *
ACTH (pg/mL)	12.44 ± 2.32	12.77 ± 2.31	222.18 ± 335.28 *	221.54 ± 333.78 *
Cortisol (ng/mL)	5.80 ± 2.22	5.6 ± 2.20	62.36 ± 40.95 *	62.72 ± 41.29 *

PMP = post-mastectomy pain group totalizing an NRS score ≥ 5; non-PMP = post-mastectomy pain group totalizing an NRS score < 5; NRS = Numerical Rating Scale; BDNF = brain-derived neurotrophic factor; ACTH = adrenocorticotropic hormone; IL-17 = Interleukin 17; IL-1β = Interleukin-1beta. Data are expressed as mean ± standard deviation. The Mann–Whitney U test and Wilcoxon test were used to compare independent groups and paired data, respectively. * = *p* < 0.01 vs. non-PMP group.

**Table 3:** Evaluation of the intensity of pain, and biomarkers of BDNF, IL-17, IL1-β, ACTH, and cortisol in the DA-PM subgroup and non DA-PMP subgroup at 3 and 6 months after surgery.

	DA-PMP *n* = 44		non DA-PMP *n* = 29	
	3 Months	6 Months	3 Months	6 Months
BDI score	31.90 ± 14.21 *	31.75 ± 14.72 *	1.55 ± 0.63	1.48 ± 0.68
GAD-7 score	13.45 ± 4.0 *	13.09 ± 3.98 *	1.41 ± 0.63	1.31 ± 0.47
NRS score (intensity of pain)	6.38 ± 1.40 *	6.22 ± 1.34 *	5.24 ± 0.45	5.31 ± 0.47
BDNF (pg/mL)	3914.33 ± 906.06 *	4031.75 ± 850.88 *	5512 ± 214.36	5504.46 ± 169.41
IL-17 (pg/mL)	94.29 ± 32.66 *	93.80 ± 30.69 *	65.68 ± 5.87	68,48 ± 7.21
IL1-β (pg/mL)	83.87 ± 14.33 *	81.91 ± 13.58 *	65.41 ± 5.88	65.09 ± 5.67
ACTH (pg/mL)	349.84 ± 382.43 *	347.58 ± 381.52 *	28.48 ± 1.44	28.03 ± 1.57
Cortisol (ng/mL)	82.33 ± 41.85 *	82.61 ± 42.45 *	32.06 ± 6.70	32.54 ± 7.16

DA-PMP = women reporting a BDI ≥ 10 and GAD-7 ≥ 5; non DA-PMP = women reporting a BDI ≤ 9 and GAD-7 ≤ 4; NRS = Numerical Rating Scale; BDI = Beck’s Depression Inventory; GAD-7 = Generalized Anxiety Disorders-7; BDNF = brain-derived neurotrophic factor; ACTH = adrenocorticotropic hormone; IL-17 = Interleukin 17; IL-1β = Interleukin-1beta. Data are expressed as mean ± standard deviation. The Mann–Whitney U test and Wilcoxon test were used to compare independent groups and paired data, respectively. * = *p* < 0.01 vs. non DA-PMP.

**Table 4:** Evaluation of physical activity by the IPAQ (International Physical Activity Questionnaire) in the DA-PMP subgroup 3 and 6 months after surgery.

IPAQ Score		
METs	DA-PMP (*n* = 44)	
	3 Months after Surgery	6 Months after Surgery
<700 (Inactive) (*n* = 20)	602.55 ± 75.55	600.95 ± 70.42
700–2509 (Adequate active) (*n* = 24)	1093.70 ± 265.46 *	1098.25 ± 266.52 *°
>2510 (Active) (*n* = 0)	N.D	N.D

IPAQ = International Physical Activity Questionnaire; DA-PMP = women reporting a BDI score ≥ 10 and GAD-7 score ≥ 5; METs = Metabolic Equivalents of Task; N.D. = not detected. Data are expressed as mean ± standard deviation. The Mann–Whitney U test and Wilcoxon test were used to compare independent groups and paired data, respectively. * = *p* < 0.01 vs. inactive. ° *p* < 0.01 vs. 3 months.

**Table 5:** Evaluation of the intensity of pain, and BDNF, IL-17, IL1-β, ACTH, and cortisol levels in the DA-PMP subgroup at 3 and 6 months after surgery, according to the physical activity score.

	IPAQ Score			
	Adequate Active DA-PMP *n* = 24		Inactive DA-PMP *n* = 20	
	3 Months	6 Months	3 Months	6 Months
NRS score (intensity of pain)	5.29 ± 0.55	5.17 ± 0.38	7.75 ± 0.85 *	7.50 ± 0.88 *
BDI score	20.16 ± 5.28	19.82 ± 5.13 °	46.0 ± 6.36 *	46.55 ± 6.02 *
GAD-7 score.	10.58 ± 2.74	10.21 ± 1.71	16.9 ± 2.07 *	16.7 ± 2.69 *
BDNF (pg/mL)	4554.06 ± 725.31	4735.25 ± 445.92	3146.67 ± 29.93 *	3187.56 ± 150.07 *
IL-17 (pg/mL)	71.50 ± 7.92	71.60 ± 2.55	121.62 ± 14.37 *	121.03 ± 12.88 *
IL1-β (pg/mL)	72.77 ± 8.91	72.37 ± 8.86	97.15 ± 5.15 *	93.88 ± 6.79 *
ACTH (pg/mL)	132.56 ± 49.55	136.20 ± 49.24	610.58 ± 443.64 *	608.70 ± 440.82 *
Cortisol (ng/mL)	53.64 ± 12.19	54.77 ± 12.19	116.75 ± 38.70 *	117.48 ± 39.39 *

DA-PMP = women reporting a BDI score ≥ 10 and GAD-7 score ≥ 5; METs = Metabolic Equivalents of Task; Active women = 700–2509 Mets: Inactive women ≤ 700 Mets. NRS = Numerical Rating Scale; BDI = Beck’s Depression Inventory; GAD-7 = Generalized Anxiety Disorders-7; BDNF = brain-derived neurotrophic factor; ACTH = adrenocorticotropic hormone; IL-17 = Interleukin 17; IL-1β = Interleukin-1β. Data are expressed as mean ± standard deviation. The Mann–Whitney U test and Wilcoxon test were used to compare independent groups and paired data, respectively. * = *p* < 0.01 vs. adequately active DA-PMP; ° = *p* < 0.01 vs. 3 months in Adequate Active DA-PMP group.

## Data Availability

The data presented in this study are available on request from the corresponding author. The data are not publicly available due to privacy.

## Supplementary Materials

The following supporting information can be downloaded at: https://www.mdpi.com/article/10.3390/life14010077/s1, Figure S1: Spearman’s correlation between Numerical rating Scale (NRS) score and BDNF, IL-17, IL-1β, ACTH, cortisol, BDI and GAD-7, in DA-PMP group, 3 months after surgery. Figure S2. Spearman’s correlation between Numerical rating Scale (NRS) score and BDNF, IL-17, IL-1β, ACTH, cortisol, BDI and GAD-7, in DA-PMP group, 6 months after surgery. Figure S3. Spearman’s correlation between Beck’s Depression Inventory (BDI) score and BDNF, IL-17, IL-1β, ACTH, cortisol and GAD-7, in DA-PMP group, 3 months after surgery. Figure S4. Spearman’s correlation between Beck’s Depression Inventory (BDI) score and BDNF, IL-17, IL-1β, ACTH, cortisol and GAD-7, in DA-PMP group, 6 months after surgery. Figure S5. Spearman’s correlation between Generalized Anxiety Disorders-7 (GAD-7) score and BDNF, IL-17, IL-1β, ACTH and cortisol in DA-PMP group, 3 months after surgery. Figure S6. Spearman’s correlation between Generalized Anxiety Disorders-7 (GAD-7) score and BDNF, IL-17, IL-1β, ACTH and cortisol in DA-PMP group, 6 months after surgery. Figure S7. Sperman’s correlation between IPAQ/NRS, IPAQ/BDI and IPAQ/GAD-7 in in DA-PMP group, 3 and 6 months after surgery. Figure S8. Sperman’s correlation between IPAQ and BDNF, IL-17, IL-1β, ACTH and cortisol in DA-PMP group, 3 and 6 months after surgery.
