# Supplementary material for: Effects of Physical Exercise and Motor Activity on Depression and Anxiety in Post-Mastectomy Pain Syndrome
Source: Life (Basel). 2024 Jan 2;14(1):77. doi: 10.3390/life14010077 (PMC10820195; doi:10.3390/life14010077)
Supplement: Supplementary file 1 [file life-14-00077-s001.zip › life-2710057-supplementary.pdf]

Figure S1. Spearman's correlation between Numerical rating Scale (NRS) score and BDNF, IL-17, IL-1β, ACTH, cortisol, BDI and GAD-7, in DA-PMP group, 3 months after surgery. DA-PMP = women reporting BDI score ≥ 10 and GAD-7 score ≥ 5; BDI = Beck's Depression Inventory; GAD-7 = Generalized Anxiety Disorders-7; BDNF = brain-derived neurotrophic factor; ACTH = adrenocorticotropic hormone; IL-17 = Interleukin 17; IL-1β = Interleukin-1beta.

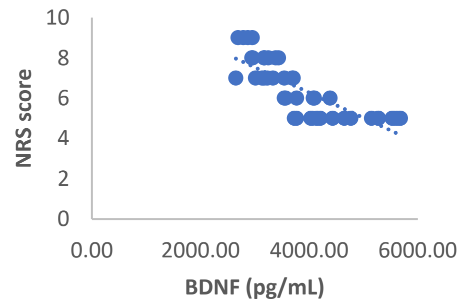

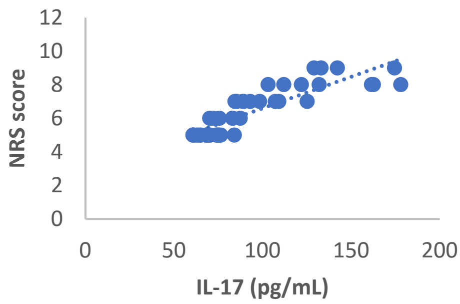

rs = -0.88222, *p* < 0.001

rs = 0.91014, *p* < 0.001

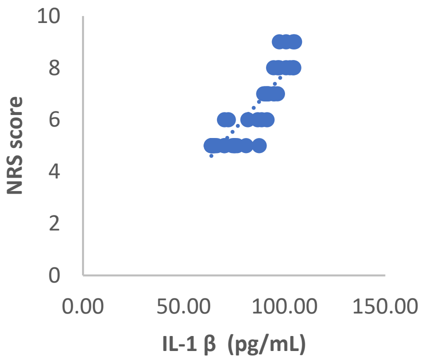

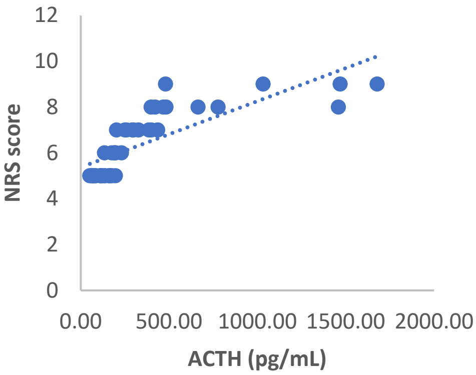

rs = 0.91732, *p* < 0.001

rs = 0.93056, *p* < 0.001

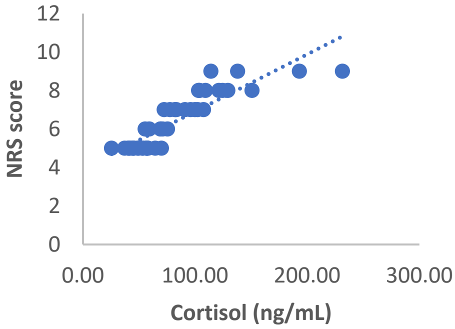

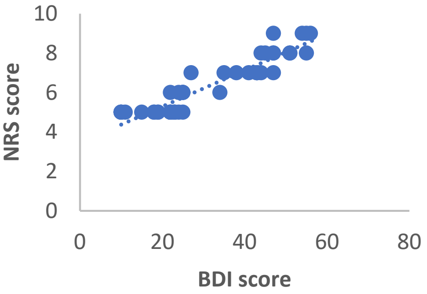

rs = 0.932, *p* < 0.001

rs = 0.93071, *p* < 0.001

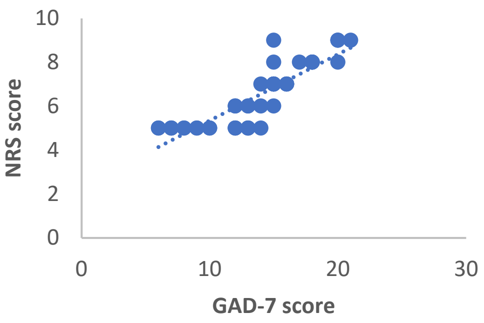

rs = 0.90087, p < 0.001

Figure S2. Spearman's correlation between Numerical rating Scale (NRS) score and BDNF, IL-17, IL-1β, ACTH, cortisol, BDI and GAD-7, in DA-PMP group, 6 months after surgery. DA-PMP = women reporting BDI score ≥ 10 and GAD-7 score ≥ 5; BDI = Beck's Depression Inventory; GAD-7 = Generalized Anxiety Disorders-7; BDNF = brain-derived neurotrophic factor; ACTH = adrenocorticotropic hormone; IL-17 = Interleukin 17; IL-1β = Interleukin-1beta.

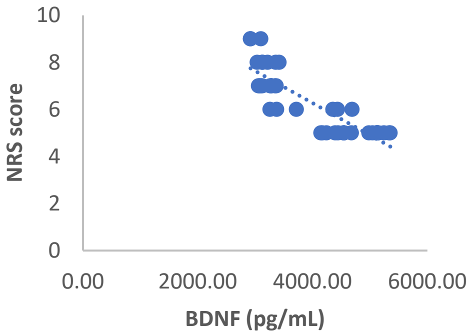

rs = -0.86477, p < 0.001

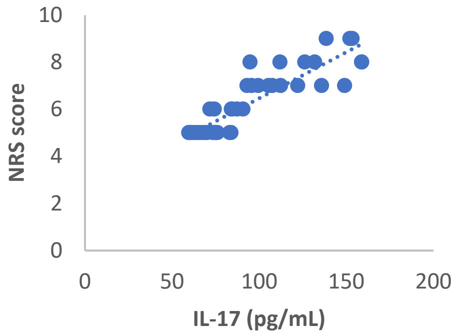

rs = 0.89196, p < 0.001

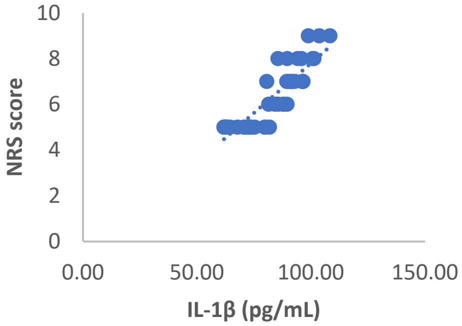

rs = 0.89816, p < 0.001

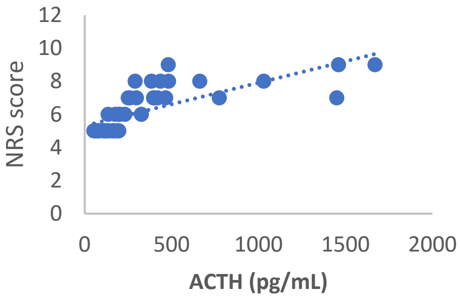

rs = 0.88876, p < 0.001

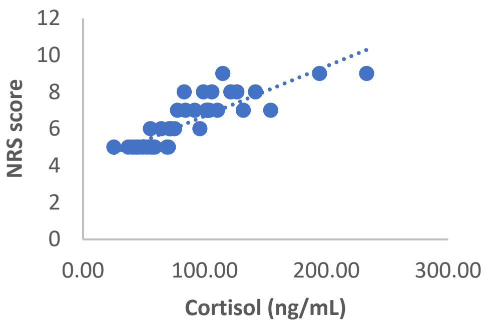

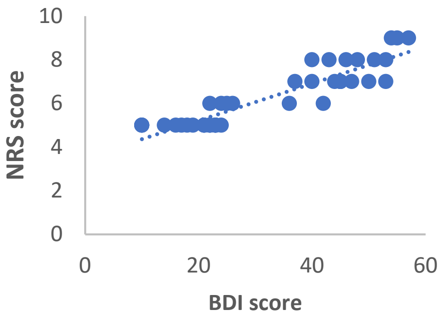

rs = 0.88764, p < 0.001

rs = 0.90454, p < 0.001

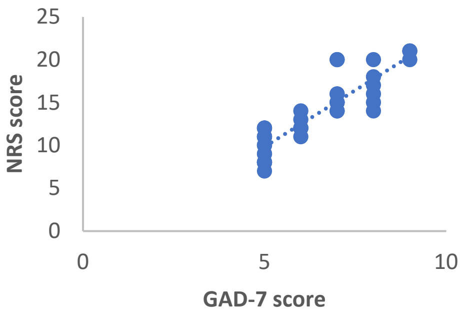

rs = 0.89787, p < 0.001

Figure S3. Spearman's correlation between Beck's Depression Inventory (BDI) score and BDNF, IL-17, IL-1β, ACTH, cortisol and GAD-7, in DA-PMP group, 3 months after surgery. DA-PMP = women reporting BDI score ≥ 10 and GAD-7 score ≥ 5; GAD-7 = Generalized Anxiety Disorders-7; BDNF = brain-derived neurotrophic factor; ACTH = adrenocorticotropic hormone; IL-17 = Interleukin 17; IL-1β = Interleukin-1beta.

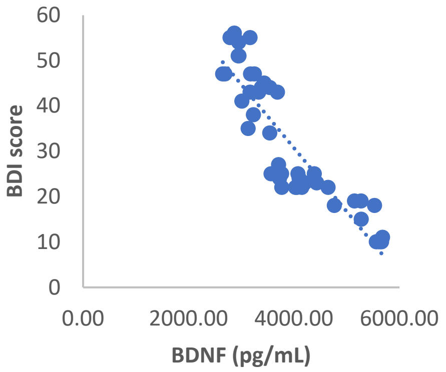

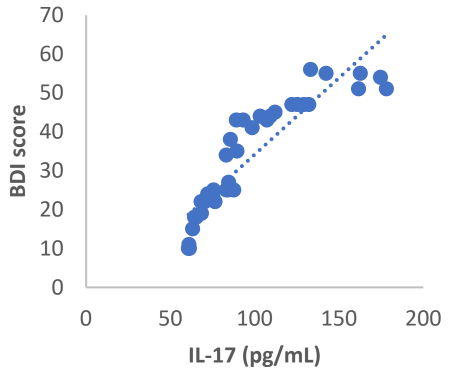

rs = - 0.93432, p < 0.001

rs = 0.98157, p < 0.001

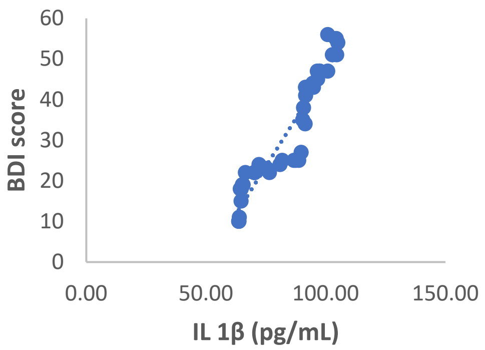

rs = 0.98892, p < 0.001

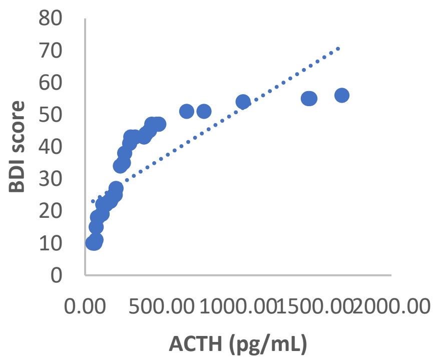

rs = 0.99802, p < 0.001

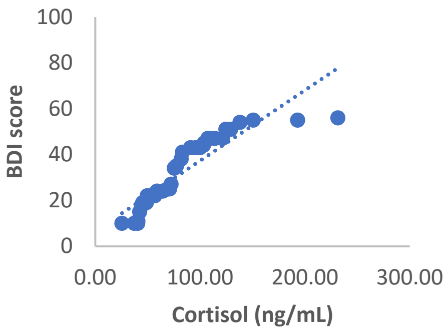

rs = 0.99799, p < 0.001

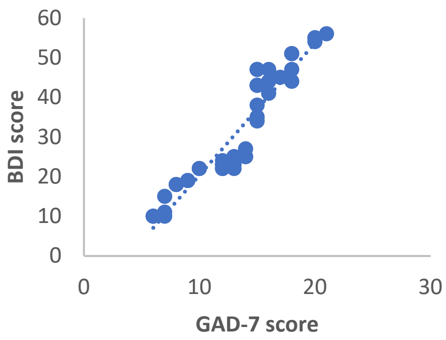

rs = 0.97374, p < 0.001

Figure S4. Spearman's correlation between Beck's Depression Inventory (BDI) score and BDNF, IL-17, IL-1β, ACTH, cortisol and GAD-7, in DA-PMP group, 6 months after surgery. DA-PMP = women reporting BDI score ≥ 10 and GAD-7 score ≥ 5; GAD-7 = Generalized Anxiety Disorders-7; BDNF = brain-derived neurotrophic factor; ACTH = adrenocorticotropic hormone; IL-17 = Interleukin 17; IL-1β = Interleukin-1beta.

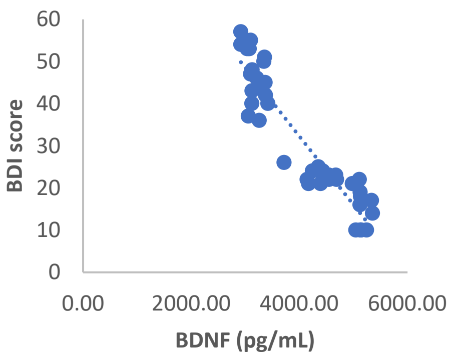

rs = -0.91489, p < 0.001

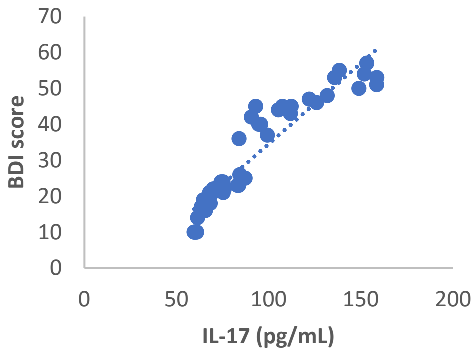

rs = 0.97357, p < 0.001

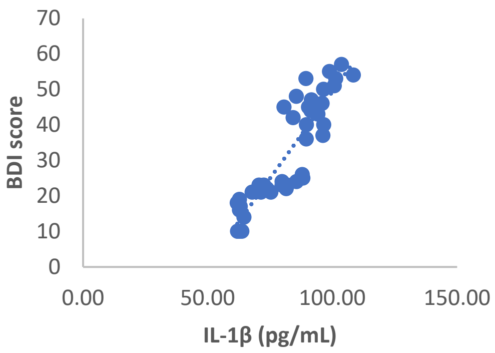

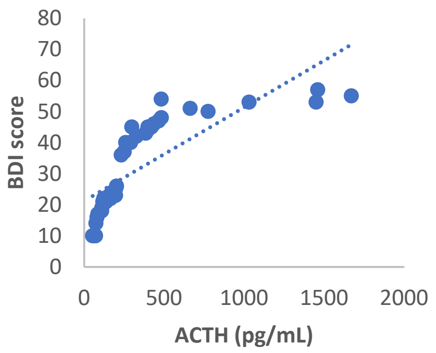

rs = 0.91386, *p* < 0.001

rs = 0.99058, *p* < 0.001

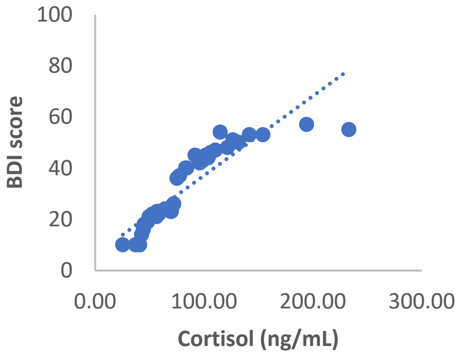

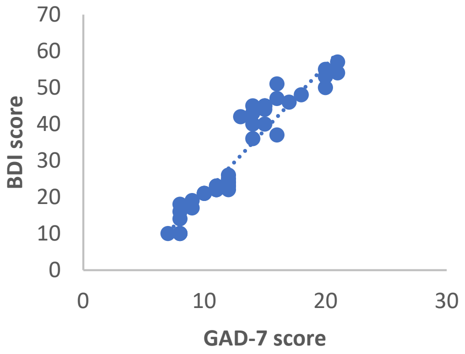

rs = 0.99002, *p* < 0.001

rs = 0.97712, *p* < 0.001

Figure S5. Spearman's correlation between Generalized Anxiety Disorders-7 (GAD-7) score and BDNF, IL-17, IL-1β, ACTH and cortisol in DA-PMP group, 3 months after surgery. DA-PMP = women reporting BDI score ≥ 10 and GAD-7 score ≥ 5; BDNF = brain-derived neurotrophic factor; ACTH = adrenocorticotropic hormone; IL-17 = Interleukin 17; IL-1β = Interleukin-1beta.

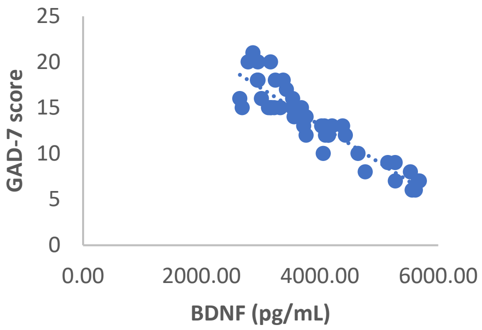

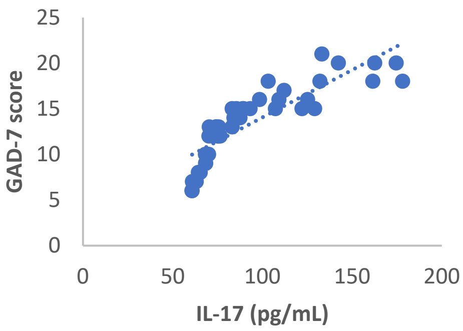

rs = -0.92469, *p* < 0.001

rs = 0.96508, *p* < 0.001

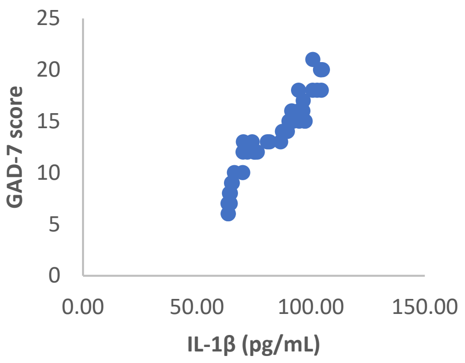

rs = 0.97011, p < 0.001

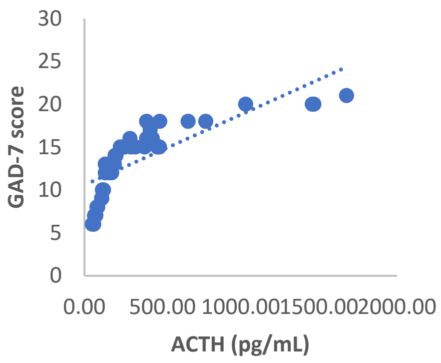

rs = 0.97574, p < 0.001

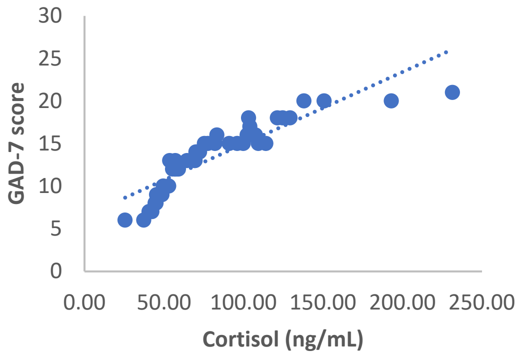

rs = 0.97603, p < 0.001

Figure S6. Spearman's correlation between Generalized Anxiety Disorders-7 (GAD-7) score and BDNF, IL-17, IL-1β, ACTH and cortisol in DA-PMP group, 6 months after surgery. DA-PMP = women reporting BDI score ≥ 10 and GAD-7 score ≥ 5; BDNF = brain-derived neurotrophic factor; ACTH = adrenocorticotropic hormone; IL-17 = Interleukin 17; IL-1β = Interleukin-1beta.

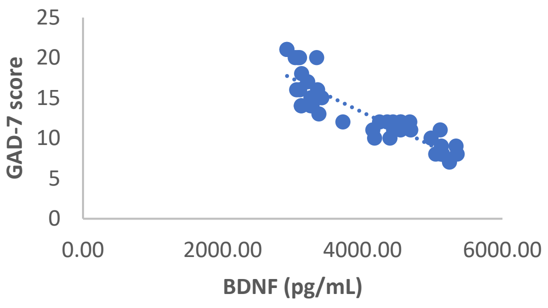

rs = -0.923, p < 0.001

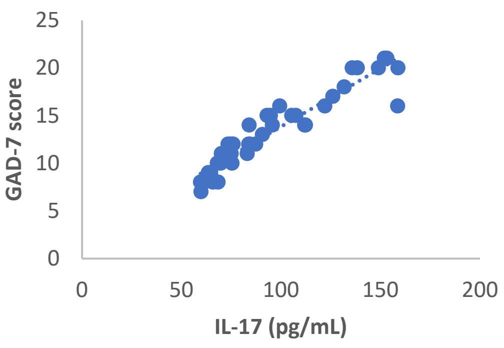

rs = 0.92559, p < 0.001

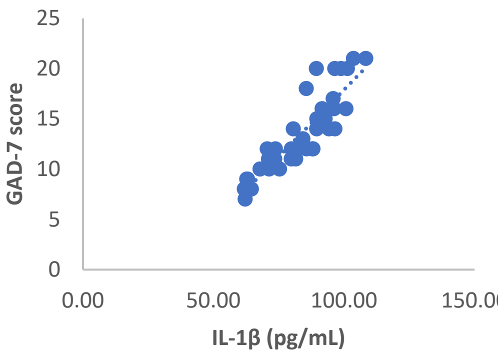

rs = 0.92559, p < 0.001

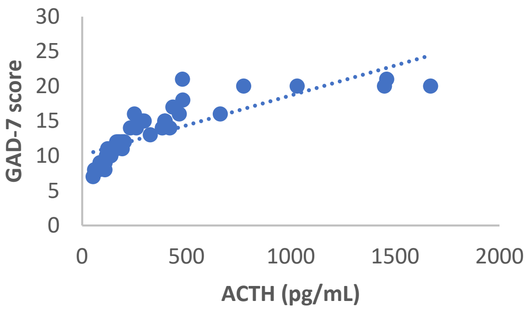

rs = 0.97136, p < 0.001

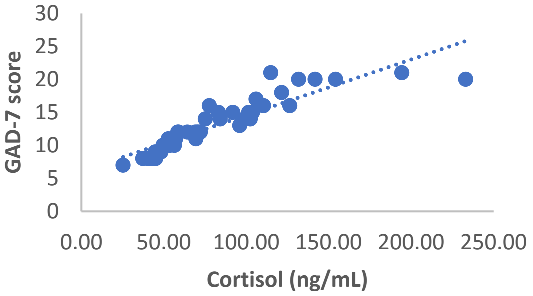

rs = 0.97136, p < 0.001

Figure S7. Sperman's correlation between IPAQ/NRS, IPAQ/BDI and IPAQ/GAD-7 in in DA-PMP group, 3 and 6 months after surgery. IPAQ = International Physical Activity Questionnaire; NRS = Numerical Rating Scale; DA-PMP = women reporting BDI score ≥ 10 and GAD-7 score ≥ 5; BDI = Beck's Depression Inventory; GAD-7 = Generalized Anxiety Disorders-7.

3 months after surgery

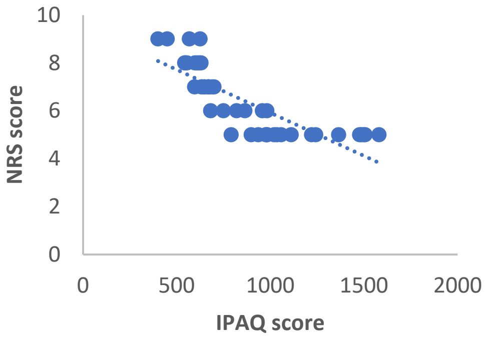

rs = -0.92328, p < 0.001

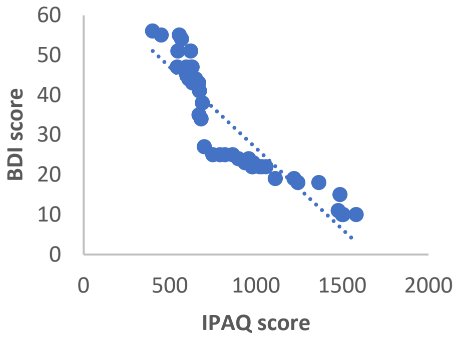

rs = -0.98736, p < 0.001

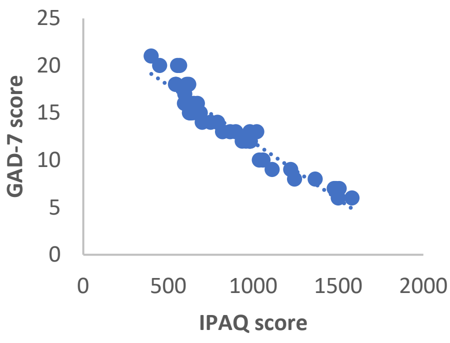

rs = -0.97649, p < 0.001

6 months after surgery

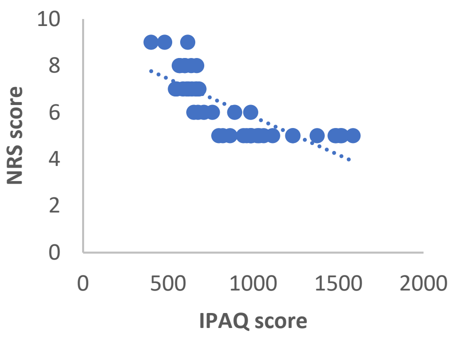

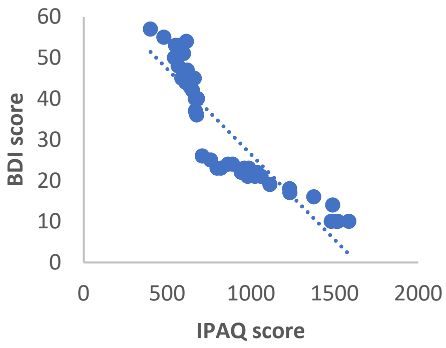

rs = -0.88621, p < 0.001

rs = -0.97879, p < 0.001

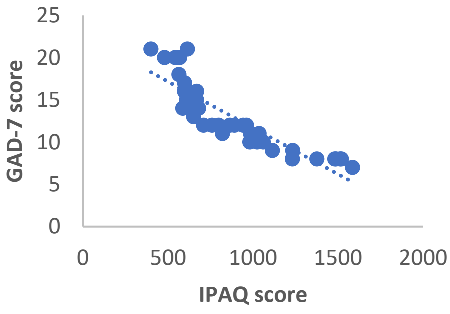

rs = -0.96285, p < 0.001

Figure S8. Sperman's correlation between IPAQ and BDNF, IL-17, IL-1β, ACTH and cortisol in DA-PMP group, 3 and 6 months after surgery. IPAQ = International Physical Activity Questionnaire; DA-PMP = women reporting BDI score ≥ 10 and GAD-7 score ≥ 5; BDNF = brain-derived neurotrophic factor; ACTH = adrenocorticotropic hormone; IL-17 = Interleukin 17; IL-1β = Interleukin-1beta.

3 months after surgery

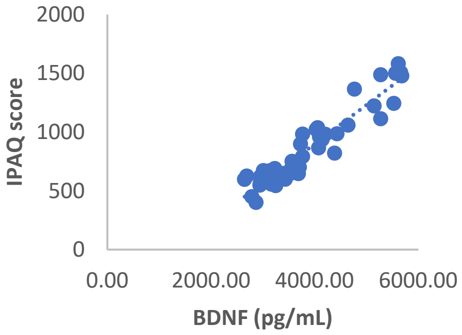

rs = 0.92741, p < 0.001

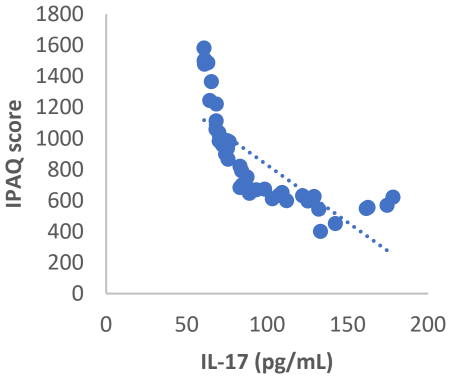

rs = -0.9753, p < 0.001

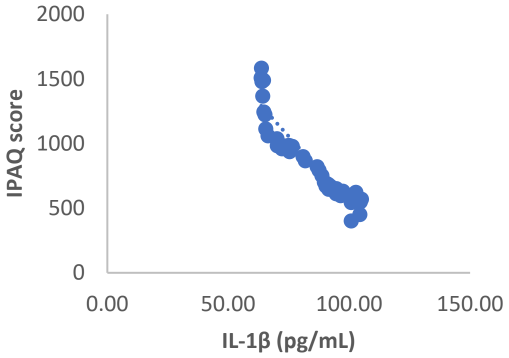

rs = -0.98407, p < 0.001

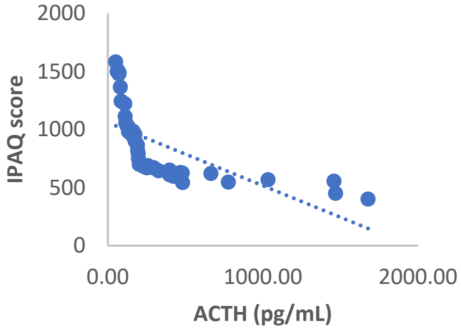

rs = -0.99091, p < 0.001

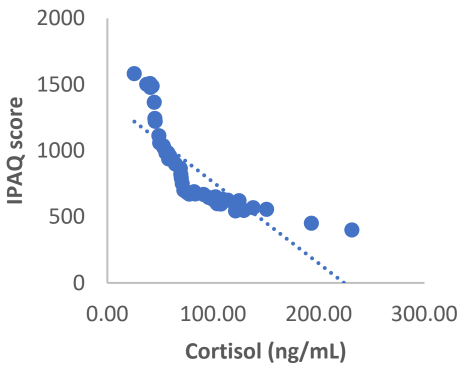

rs = -0.99158, p < 0.001

6 months after surgery

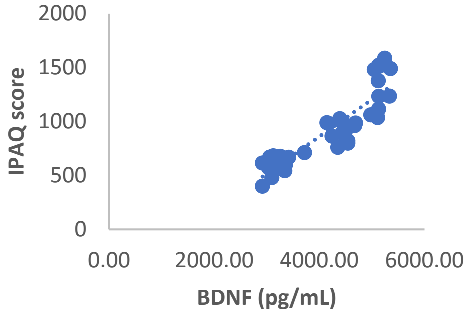

rs = 0.90672, p < 0.001

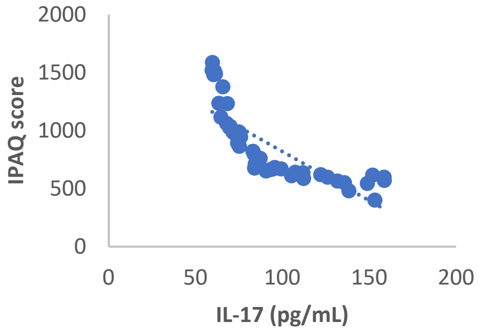

rs = -0.97583, p < 0.001

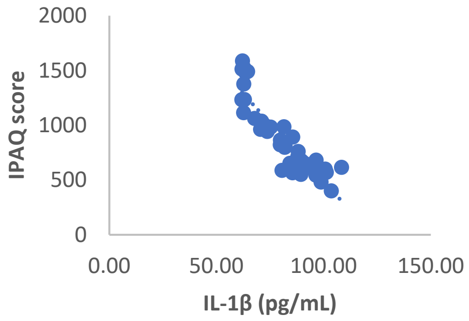

rs = -0.88928, p < 0.001

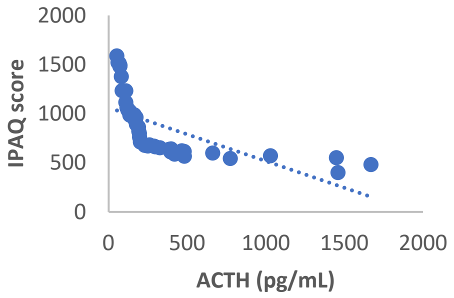

rs = -0.99309, p < 0.001

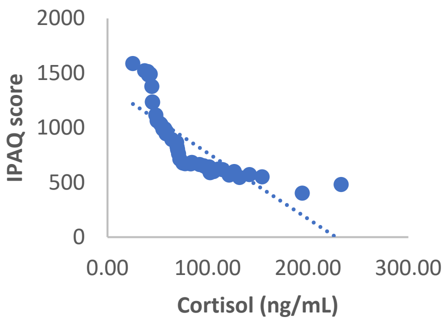

rs = -0.99281, p < 0.001
